# Terry's nails

**DOI:** 10.1002/ccr3.2671

**Published:** 2020-01-17

**Authors:** Sreenath Meegada, Rajanshu Verma

**Affiliations:** ^1^ UT Health East Texas Christus Good Shepherd Medical Center Longview TX USA; ^2^ UTHSC College of Medicine Memphis TN USA

**Keywords:** cirrhosis, congestive heart failure, renal failure, Terry's nails

## Abstract

Terry's nails can be manifested in systemic diseases like cirrhosis, congestive heart failure, diabetes mellitus, renal failure, and other conditions which emphasizes the importance of physical examination in every clinical encounter.

## Graphical abstract

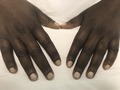

## 1 INTRODUCTION

**Figure 1:**
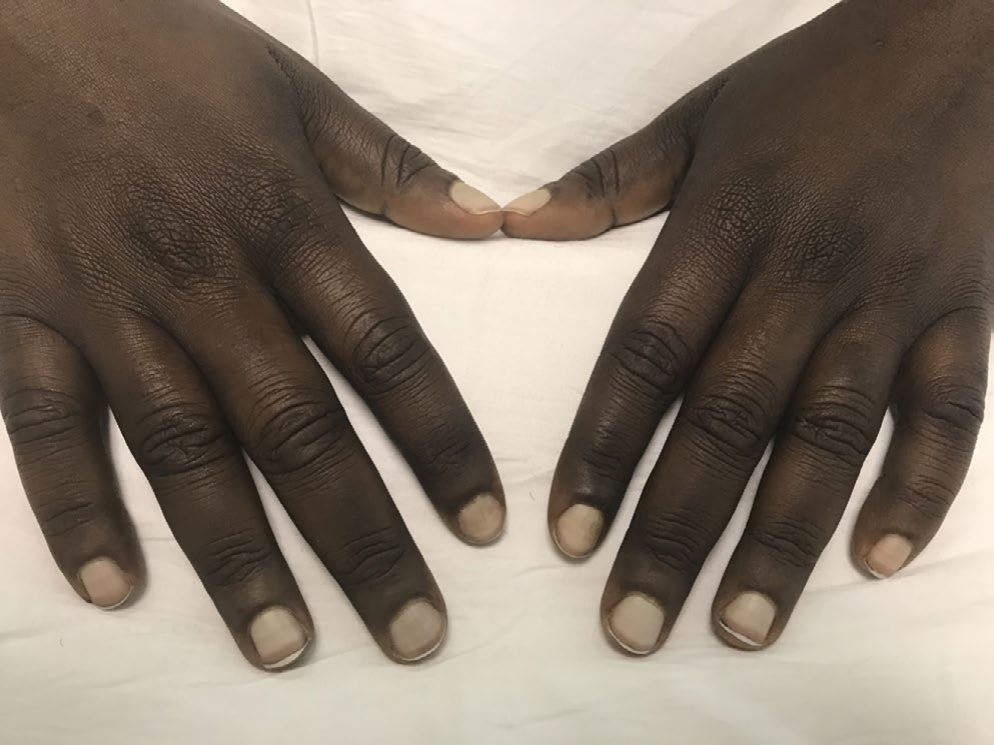
Terry's nails in a patient with Cirrhosis from primary sclerosing cholangitis

A 26‐year‐old man with decompensated cirrhosis due to underlying primary sclerosing cholangitis complicated by episodes of recurrent cholangitis, variceal hemorrhage, and hepatic encephalopathy in the past presented to the hospital with recurrent hepatic encephalopathy and hypokalemia. On examination, he was found to have whitish “ground glass” discoloration of the nail beds with absence of lunula and presence of distal normal pink zone (Terry's nails) (Figure 1) Terry's nails were initially described by Dr Richard Terry in 1954 in patients with cirrhosis.^1^ They may also be found in congestive heart failure, chronic renal failure, and other conditions.^2^, ^3^, ^4^ His encephalopathy was corrected with administration of lactulose, and low potassium was replaced. Given his recurrent episodes of cholangitis, he was listed for liver transplantation and is currently awaiting a liver transplant.

## AUTHOR CONTRIBUTIONS

SM: wrote the manuscript, contributed to the article, and proofread the entire article prior to submission. RV: took care of the patient, collected clinical data, conceptualized the article, and did final proofreading of the submission.
